# Effect of resilience training on stress, hope and psychological toughness of mothers living with mentally and physically disabled children

**DOI:** 10.1186/s12887-024-04828-6

**Published:** 2024-05-22

**Authors:** Pegah Sharifian, Zeinab kuchaki, Mahnaz shoghi

**Affiliations:** 1 Nursing & Midwifery Care Research Center, School of Nursing & Midwifery, Pediatric and Neonatal Intensive Care Nursing Department, Iran University of Medical Sciences, Tehran, Iran; 2 Critical Care Nursing Department, School of Nursing and Midwifery, Ilam University of Medical Sciences, Ilam, Iran

**Keywords:** Disability, Resilience, Stress, Hope, Resilience

## Abstract

**Introduction:**

Welfare and rehabilitation centers prioritize the welfare of children over the mental and physical well-being of mothers. The present study aimed to determine the impact of resilience training on stress, hope, and psychological toughness of mothers living with mentally and physically disabled children.

**Materials and methods:**

This intervention study was conducted in the Hamadan (Iran) Welfare and Rehabilitation Center in 2023. To this end, 70 parents of children with mental and physical disabilities were randomly selected and then randomly assigned to two control and intervention groups. In the intervention group, 9 resilience training sessions were conducted, each lasting 60 min. These meetings were held weekly at the welfare and rehabilitation center. The resilience training included three components: (1) exploring the concept of resilience within families and the attributes of individuals with high resilience, (2) examining internal and external factors that influence resilience, and (3) studying the strategies for enhancing family resilience. No intervention was performed in the control group. Data collection was done using parental stress, hope, and psychological toughness questionnaire. The mothers of both groups completed the above questionnaires both before and one month after the intervention. Data analysis was performed using chi-square (χ2), Kruskal-Wallis, and t-test with SPSS software (version 23) at a significance level of 0.05.

**Results:**

Before the intervention, there was no statistically significant difference in parental stress between the two groups (*p* = 0.370). However, after the intervention, the difference between the two groups became statistically significant (*p* = 0.001). Similarly, there was no significant difference in parents’ hope before the intervention (*p* = 0.452), but a significant difference was observed after the intervention (*p* = 0.001). Besides, parental psychological toughness was not significant before the intervention (*p* = 0.179) but became significant after the intervention (*p* = 0.000).

**Conclusion:**

Based on the results, resilience training reduced parental stress and increased hope and resilience in mothers of the test group. Therefore, resilience training is recommended to lower parental stress and increase the hope and psychological toughness of mothers of mentally and physically disabled children.

## Introduction

The restrictive and chronic conditions experienced by children often result in stress and psychological challenges for both the individual and their family [1]. In this respect, having a child with mental and physical challenges perpetuates an ongoing sense of discordance between parents and their child’s disability [2]. Parents of children with developmental disabilities experience mental sicknesses such as depression, frustration, and anxiety compared to parents of children without any mental disabilities [3, 4].

While the majority of studies concerning children with intellectual and physical disabilities have predominantly centered on parental involvement, it is imperative to recognize the pivotal role of mothers as primary caregivers and facilitators of their children’s care and rehabilitation. Their contribution is indispensable across different phases of the rehabilitation journey, often placing them under significant emotional strain within the caregiving team. Consequently, welfare and rehabilitation centers concentrate on the welfare of children rather than prioritizing mothers’ mental and physical well-being [5, 6, 7]. One of the most important reasons for the vulnerability of mothers is that they spend more time with their children compared to other family members and fathers. Besides, mothers feel more responsible in raising their children and are often involved in their children’s problems [8]. Research has shown that the negative effects of having a weak or disabled child cause tension and pressure on family members, especially the mother [9].

Mothers of children with mental and physical disorders are under stress, such as taking care of the child and controlling and raising the child, which may gradually result in a feeling of burnout and fatigue [10]. Therefore, psychological toughness and resilience are effective factors in the mental functions of mothers. These factors can improve their ability to deal with emotional and psychological problems by improving their creation, behavior, and thinking [11].

Resilience is an essential concept representing that individuals with a disabled child can maintain their mental health [12]. Resilience also refers to positive outcomes after experiencing adversities, positive and effective performance under adverse conditions, and recovery after a serious mental or physical injury [13]. Previous studies have reported that resilience increases a person’s ability in difficult situations, but no significant relationship has been detected between resilience and quality of life [14, 15].

In a similar study, Wu et al. reported that social support, hope, and resilience significantly affect the quality of life [16]. Also, research has reported a direct relationship between resilience and toughness [17]. Therefore, conducting studies on the relationship of resilience interventions with stress control, hope, and psychological toughness can help improve the activity and performance levels of mothers of children with mental and physical disabilities. Regarding the mentioned points, the present study aimed at determining the impact of resilience training on stress, hope, and psychological toughness of mothers living with mentally and physically disabled children.

## Materials and methods

This semi-experimental pre-test and post-test study was conducted in the Hamadan (Iran) Welfare and Rehabilitation Center in 2023. The case samples included 70 mothers living with mentally and physically disabled children referred to the welfare and rehabilitation centers of Hamadan city.

### Inclusion and exclusion criteria

Inclusion criteria were the mother’s minimum age of 20 and maximum of 50 years, reading and writing literacy, having a child with a definite diagnosis of physical or mental disability, and at least one year passing since the diagnosis. Exclusion criteria included not agreeing to participate in the study for any reason and not attending training classes for more than two sessions.

### Sample size

The sample size was calculated based on the recommendations of Kaboudi et al. [18]. The first type of error level was considered to be 0.05, and the power of the test was 90%.

$$\begin{array}{l}n = \frac{{{{(Z_{1 - \alpha /2}^2 + {Z_{1 - \beta }})}^2} \times [{\sigma _1}^2 + {\sigma _2}^2]}}{{{{({\mu _1} - {\mu _2})}^2}}} = \\\frac{{{{(1.96 + 0.842)}^2} \times [{{11.34}^2} + {{16.50}^2}]}}{{{{\left( {10} \right)}^2}}} = 31\end{array}$$

In each group of 31 people and with a drop of 10%, the sample size was considered to be 35 individuals. Therefore, the sample size was estimated to be 70 individuals.

### Tools

This study used a demographic questionnaire, parental stress scale short form questionnaire, hope questionnaire, and Kubasa psychological toughness questionnaire for collecting data. The demographic questionnaire included the age of the mother, father, and child, the mother and father’s education, the mother and father’s occupation, the family’s economic status, the child’s gender, and the number of children. The questionnaire of the short form of the parental stress scale was directly extracted from the long form of this scale prepared by Abdin (1983) in response to the needs of clinical professionals and researchers. The scale was prepared based on the theory that the general stress that parents experience is a function of the child’s behavioral characteristics, the parents’ personality traits, and the psychological pressures of the family environment, which are directly related to the role of parents. The short form of the Parental Stress Scale consisted of 36 questions with the same wording as in the original long form of 101 questions. Hosseini Qomi et al. (19) determined reliability through a test-retest of 531 mothers with a time interval of six months and an overall stress reliability coefficient of 84%. Cronbach’s alpha of parental stress scale in this study was 0.80.

The hope questionnaire is a 12-item questionnaire introduced by Schneider et al. (1991) to evaluate the level of hope in people. This questionnaire is designed for people aged 15 years and above, and its scoring method is based on the 5-option Likert scale as follows: I completely agree (= 5), I agree (= 4), I have no opinion (= 5), I disagree (= 5), and I completely disagree (= 5). The overall score of the questionnaire was calculated by summing the scores of each question. Higher scores indicate more hope in the respondent, so scores between 12 and 24 indicate a low level of hope, scores between 24 and 36 show an average level of hope for life, and scores above 36 represent a high level of hope [20]. In Iran, the validity and reliability of this questionnaire were checked using the internal consistency method, and Cronbach’s alpha coefficient was 0.89 [21, 22]. Cronbach’s alpha of the hope questionnaire in this study was 0.83.

The Kubasa Psychological Toughness Questionnaire was designed by Kubasa et al. (1982) to evaluate an individual’s psychological toughness. The short form of this questionnaire consisted of 20 items on a 4-point Likert scale (never, rarely, sometimes, and often), which is summarized and analyzed as a score between 0 and 3. The scoring method in this questionnaire is as follows: never (= 0), rarely (= 1), sometimes (= 5), and often (= 5). This scale includes 3 scales of control, commitment, and struggle. The overall score of the questionnaire is obtained by summing the score of each question. A higher score indicates more toughness in the respondent and a lower score indicates less toughness. Cronbach’s alpha coefficient for the internal consistency of this questionnaire was 0.67 [23]. Cronbach’s alpha of psychological toughness questionnaire in this study was 0.79.

### Intervention

After obtaining the necessary permissions, the researcher referred to the welfare and rehabilitation centers in Hamadan and introduced himself as the supervisor and manager of the center. Then, the mothers of children with mental disorders and physical disabilities were selected as samples. The research objectives were explained, and the participants submitted a written consent form. All participating mothers in the experimental group completed the Parental Stress Scale, Hope Scale, and Psychological Resilience Scale questionnaires. Next, in the experimental group, the researcher held nine 60-min resilience training sessions, one session per week (approximately 2 months). The sessions were held in a classroom located at the welfare and rehabilitation center. The resilience training covered three areas: (1) the concept of resilience for families and the characteristics of individuals with high resilience, (2) internal and external factors influencing resilience, and (3) the ways to increase family resilience (Table 1). One month after the intervention, in the experimental group, the participants were asked to complete the Parental Stress Scale, Hope Scale, and Psychological Resilience Scale questionnaires again. In the control group, the participating mothers first completed the Parental Stress Scale, Hope Scale, and Psychological Resilience Scale questionnaires. Next, in this group, without any intervention, the same questionnaires were again completed by the mothers two months later.

**Table 1:** Resilience skill training protocol in the present study [18]

Session number	Session content
**First session**	Pre-intervention - providing guidance for the participation of members and explaining how to do the work: the researcher introduces himself, introduces the members, explains the general direction of the meetings to the members
**Second session**	An introduction to the general framework of the discussion: (1) Define resilience, (2) Introduce the characteristics of resilient people: (1) Joy (2) Wisdom and insight, 3) Humor, 4) Sympathy, 5) Rational adequacy, 6) Purposefulness in life, and 7) Stability.
**Third session**	Solution: Understanding the unpleasant situations in life and increasing coping and tolerance in personal life. Objective: Familiarity with internal support factors 1) The concept of optimism 2) Self-esteem 3) Source of control Solution: Recognizing and emphasizing talents and interests and willingness to use them
**Fourth Session**	Purpose: Familiarity with external support factors 1) Social support system 2) Individual responsibility and acceptance of important roles) Solution: the sense of belonging and value and willingness to participate
**Fifth meeting**	Purpose: Familiarity with the methods of providing resilience; establishing and maintaining relationships with others, a framework for parental stress and problem acceptance
**Sixth session**	Purpose: to continue providing resilience; purposefulness and hope for the future, acting
**Seventh session**	Purpose: to continue providing resilience; self-awareness and self-confidence development
**Eighth session**	Purpose: to continue providing resilience; self-care
**Ninth session**	Purpose: conclusion and implementation of the post-intervention

## Statistical analysis

Descriptive statistics were used to describe the data, and chi-square (χ2) and t-test were used to analyze the data using SPSS software (version 23) at a significance level of 0.05.

## Results

Regarding the qualitative demographic characteristics of the participants, the education level of the mothers in the intervention group (47.3%) and the control group (45.6%) was higher in elementary school. The studied children in both groups were mostly male (51.7%). The results of the χ2 test showed that both groups were similar in terms of all demographic variables. Regarding the quantitative data of the present study, the majority of participating mothers in both experiment and control groups were 29–42 years old. The number of family members in the experiment and control groups was 4.

**Table 2:** Intra-group comparison of parental stress, hope, and psychological toughness before and after the intervention between the intervention and control groups

Group		Mean	SD	*P*-value
parental stress	intervention	110.13	17.54	0.000
		70.43	14.12	
	control	102.89	11.56	0.254
		105.98	16.45	
Hope	intervention	39.56	3.76	0.000
		45.87	2.98	
	control	39.77	3.55	0.031
		43.59	3.89	
psychological toughness	intervention	51.56	12.43	0.000
		72.34	6.54	
	control	53.79	9.90	0.155
		50.16	6.87	

* the intra-group comparison based on the paired t-test

Table 2 presents the results of the intra-group comparison of parental stress before and after the intervention in the experimental group. As can be seen, there is a significant difference between the interventions, and resilience training was able to reduce parental stress in mothers of children with cancer (*p* = 0.000). However, in the control group, the intra-group comparison of parental stress before and after the intervention showed no significant difference (*p* = 0.254). The intra-group comparison of hope before and after the intervention in the experimental group showed a significant difference and resilience training was able to increase hope in mothers of children with cancer (*p* = 0.000). Moreover, in the control group, the intra-group comparison of hope before and after the intervention revealed a significant difference (*p* = 0.031). The intra-group comparison of psychological resilience before and after the intervention in the experimental group showed a significant difference, and resilience training could increase psychological resilience in mothers of children with physical and mental disabilities (*p* = 0.000). However, in the control group, the intra-group comparison of psychological resilience before and after the intervention showed no significant difference (*p* = 0.155).

**Table 3:** Intergroup comparison of parental stress, hope, and psychological toughness before and after the intervention between the intervention and control groups

Group		Intervention		Control		T	Df	*P*-value
		Mean	SD	Mean	SD			
parental stress	Before intervention	110.13	17.54	102.89	11.56	8.18	68	0.370
	After intervention	70.43	14.12	105.98	16.45	-0.437	68	0.001
hope	Before intervention	39.56	3.76	39.77	3.55	-4.67	68	0452
	After intervention	45.87	2.98	43.59	3.89	-2.87	68	0.001
psychological toughness	Before intervention	51.56	12.43	53.79	9.90	-6.43	68	0.179
	After intervention	72.34	6.54	50.16	6.87	0.987	34	0.000

* the intra-group comparison based on t-test

As shown in Table 3, Before the intervention, there was no statistically significant difference in parental stress between the two groups (*p* = 0.370). Nevertheless, after the intervention, the difference between the two groups became significant (*p* = 0.001). Similarly, there was no significant difference in parents’ hope before the intervention (*p* = 0.452), but a significant difference was observed after the intervention (*p* = 0.001). Additionally, parental psychological toughness was not statistically significant before the intervention (*p* = 0.179), but became significant after the intervention (*p* = 0.000).

**Table 4:** Mean Difference of Hope, Parental Stress, and Psychological toughness Scores before and after Intervention between the Experimental and Control Groups

Group	Intervention				Control			
	Minimum	Maximum	Mean	SD	Minimum	Maximum	Mean	SD
Hope	-7	14	3.37	4.23	-12	18	2.74	5.75
Parental Stress	91-	10	-28.55	19.31	-87	51	3.26	24.77
psychological toughness	2	54	19.78	14.31	-38	32	4.20	15.50

* the intra-group comparison based on the Paired Samples Test

As shown in Table 4, the average hope scores in the intervention and control groups were 3.37 and 2.58, respectively. These findings suggest that hope increased by an average of 3.37 and 2.58 units in the intervention and control groups, respectively, and both groups experienced an improving trend in the hope variable. The average parenting stress score in the intervention group was − 28.55, while in the control group was 3.26. These results indicate that in the intervention group receiving resilience training, parental stress was reduced by 28.55 units, while this level increased by an average of 3.26 units in the control group without any intervention. Thus, parental stress in this group was increased. The mean score of psychological toughness increased by 19.78 units on average in the experimental group. Meanwhile, in the control group, the mean resilience score of mothers decreased by 4.20 units on average, indicating a decrease in resilience in this group.

## Discussion

This study aimed to examine the effects of resilience training on the stress, hope, and psychological toughness levels of mothers who have children with mental and physical disabilities. The results showed no significant difference between the groups in terms of parental stress scores before the intervention, but there was a significant difference after the intervention. These results are consistent with the findings of Kore and Venkatraman [24], Hosseini-Ghomi and Jahanshahi [25], Lida et al. [26], Moghimi et al. [27], Mohammadi et al. [28], Aslani et al. [29], Motaghi et al. [30], Kaboudi et al. [18], and Hosseini-Ghomi et al. [19] studies.

Kore and Venkatraman reported that parents of children with mental disabilities with a higher level of hope showed a lower score in perceived stress compared to parents with a lower level of hope [24]. In other words, the factors increasing hope in the parents of children with mental disabilities can simultaneously lower their stress levels. Therefore, methods of increasing hope can be used to reduce the perceived stress of parents of children with mental disabilities. Moreover, Hosseini Qomi and Jahanbakhshi reported that resilience training programs and the formation of counseling and support groups can be used as part of intervention programs to reduce stress and improve the mental health of mothers of children with mental disabilities [25].

Lida et al. reported a statistically significant difference among the average scores of stress, anxiety, and depression before and after the intervention in the research samples. As a result, parental training is effective in improving the coping style of mothers of children with autism spectrum disorder and reducing their levels of depression and anxiety [26]. In another study, Moghimi et al. found that resilience training through text messages can reduce the stress of mothers of children with intellectual disabilities [27]. Based on the mentioned study, it can be concluded that mothers of children with mental health problems suffer from various disorders, such as stress, which can have long-term negative effects on other aspects of their lives. Therefore, using minimal education and support allows for minimizing this disorder in the mothers of children with mental health problems. Elsewhere, Mohammadi et al. reported that the effect of resilience training on reducing parenting stress was significant and its effect was stable after two months of training [28]. Therefore, it can be concluded that systematic and planned education in the long term can have significant and lasting effects on reducing parental stress. Hence, it is necessary to include such training programs in parenting education programs for parents of children with mental and physical disabilities. In this regard, Aslani et al. found that the resilience level and improvement of the mother-child relationship in mothers who underwent this treatment program were significantly higher than in mothers who did not receive treatment, and parental stress was reduced in the intervention group [29]. Training programs such as resilience training reduce parental stress and improve the parent-child relationship by strengthening parents, empowering mothers to solve the challenges ahead, and promoting constructive thinking. In this respect, Motaghi et al. reported that resiliency training for mothers is effective in reducing their stress, and resiliency training can result in strengthening abilities such as managing negative emotions, problem-solving, and constructive thinking in mothers, thereby improving the relationship between parents and children [30].

Kaboudi et al. reported that mothers trained in resilience skills made significant progress in increasing coping styles and reducing parental stress compared to mothers in the control group [18]. In this regard, Hosseini Qomi et al. concluded that resilience training significantly reduced stress and increased mothers’ resilience [19]. Therefore, it is inferred that using resilience training is an effective method for reducing parental stress, especially for mothers of autistic children. In all the above studies, it was also reported that resilience training reduced the stress of parents of children with various mental and cognitive disorders. Furthermore, the studied parents experienced less stress after receiving resilience training.

The findings of the present study indicated that the difference in the average score of hope before the intervention in the intervention and control groups was not significant, but it was significant after the intervention. These findings are consistent with those of Khosrobeigi et al. [31], Martin et al. [32], Rafiepoor [33]. , Momeni [34]. , Reza Zadeh et al. [35], Akbari et al. [36], and Kanwal M, Asad [37]. It was anticipated that the resilience training intervention would increase the hope of mothers in the intervention group. However, in the control group, the hope level in the mothers who did not receive any intervention increased compared to the pre-intervention state. This result indicated that mothers of children with mental and physical disabilities are encouraged by medical staff and other mothers with similar experiences. Additionally, according to cultural beliefs, they are encouraged to have hope and rely on a higher power, which may be an effective factor in increasing the control group’s hope level. However, comparing the hope levels of mothers in the intervention and control groups revealed that the average hope score among mothers in the intervention group was higher than that of mothers in the control group. In this regard, Khosrobeigi et al. concluded that teaching self-compassion had a significant effect on increasing resilience and reducing hopelessness among parents of children with cancer [31]. Martin et al. also showed that changes in the level of gratitude and hope cause changes in depression; however, no change was observed in the anxiety score [32]. Rafiepoor et al. reported that hope therapy could improve the quality of life of mothers with disabled children and also showed that hope therapy acts as an effective step in improving their mental state and parent-child relationship [33]. The results of these studies suggest that parents of children with mental disorders and physical disabilities generally face frustration and other problems in the path of their child’s growth and development. Their resilience and hope can be increased with support and training. This return of hope can double their strength to continue the path, support their child, and increase their hope for the future.

Moreover, Momeni et al. reported that the average score of expectancy and psychological well-being of mothers with children with special learning disabilities in the intervention group increased compared to the control group [34]. Therefore, it can be said that resilience training was effective on the life expectancy and psychological well-being of mothers with children with special learning disabilities. In this regard, Reza Zadeh et al. concluded that providing educational and therapeutic interventions based on positive psychology can be effective in promoting hope and resilience against stress in mothers with mentally disabled children [35]. Akbari et al. reported that the implementation of resilience training programs can effectively reduce aggression and increase happiness in students; hence, it is useful to add these programs to the university curriculum [47]. Kanwal and Asad found a positive and significant relationship between resilience and hope [37]. All these studies showed that resilience training was able to give hope to mothers of children with various mental and cognitive disorders, and the studied parents experienced a higher hope level after receiving resilience training.

The present study’s findings showed that the difference in the average score of psychological toughness in the intervention and control groups was not significant before the intervention. However, it was significant after the intervention, which is in agreement with the findings of Dunseth [38], Sabz Menesh Jafari et al. [39], Mohammadi Hasil et al. [28], Sanei Menesh et al. [40], Salehian and Sarvari [41], and Khodabakhshi Kolai [42]. Dunst et al. concluded that family resilience is an internal resource that simultaneously has stress-inhibiting and health-enhancing effects on parenting and family functioning [38]. Sabz Menesh Jafari et al. also found that family resilience training is a suitable intervention to improve psychological toughness in mothers of disabled children and should be used in rehabilitation clinics and family training centers [39]. Therefore, another advantage of resilience training is increasing the resilience of mothers in facing situations and challenges. These results double the importance of the role of nurses involved in caring for children with mental and physical disabilities. Basic and planned trainings, such as resilience, make mothers tougher and more resilient in dealing with and solving the challenges. In this regard, Mohammadi et al. reported that using a resilience training program can effectively increase resilience and reduce the perceived stress level of parents of students with disabilities [28]. Moreover, Sanieemanesh et al. showed that group counseling based on acceptance and commitment therapy improves marital stress and psychological toughness of mothers of children with severe and multiple disabilities [40]. These findings are consistent with those of Salehian & Sarvari and Khodabakhshi-Koolaee et al. [41, 42].

The findings of the above studies highlighted the role of resilience training in reducing the stress level of mothers and families, thereby enhancing the toughness of mothers to deal with unfortunate and challenging situations. Therefore, it can be concluded that resilience training can increase the psychological toughness of mothers of children with various mental and cognitive disorders. In all studies, the parents experienced higher psychological toughness after receiving resilience training.

The general findings of this research showed that after resilience training, the parents’ stress levels decreased, and the level of hope and psychological toughness of mothers in the intervention group increased. It seems that when a mother learns resilience, her stress level decreases. Also, in the presence of a lower amount of stress, the hope level increases and the mother shows more toughness against the challenges ahead. Consistent with these findings, Mohammadi et al. reported that the effect of resilience training on reducing parental stress was significant, and its effect remained stable after two months. Therefore, resilience training can lead to long-term reduction in the level of stress in the studied mothers. As a result, lowering stress levels could increase the levels of hope and resilience in mothers. Therefore, resilience training can be used as an effective factor in helping mothers of children with mental and physical disabilities to cope with various challenges of the disease. Additionally, Aslani et al. found that the level of resilience and improvement in mother-child relationships in mothers who received this treatment program was significantly higher compared to those who did not receive treatment, and parental stress decreased in the intervention group [29].

Moreover, Motaghi et al. showed that resilience training for mothers effectively lowers their stress levels and can strengthen their skills, such as negative emotion management, problem-solving, and constructive thinking, improving parent-child relationships. Therefore, according to the findings of the above studies, resilience training can be used not only to increase levels of hope and reduce maternal stress but also to improve parent-child relationships and enhance constructive thinking for facing future challenges.

Kaboudi et al. showed that mothers trained in resilience skills made significant progress in increasing coping styles and reducing parental stress compared to mothers in the control group [18]. Momeni et al. found that the average score of life expectancy and psychological well-being of mothers with children with special learning disabilities of the intervention group increased compared to the control group [34]. Don Seth also concluded that family is an internal resource with simultaneous stress-relieving and health-enhancing effects on the functioning of parents and the family [38]. In this regard, Jafari et al. reported that family resilience training is a suitable intervention to improve psychological toughness in mothers of disabled children and should be used in rehabilitation clinics and family education centers [43]. Therefore, resiliency training can be used to empower parents against difficulties and face the challenges of their children’s growth and development. Moreover, these results are consistent with those reported by Salehian and Sarvari. They reported that toughness equips a person with a shield to deal with stressful situations, thereby lowering the level of anxiety and depression. In addition, it enables a person to look at events more optimistically by activating problem-based coping strategies in stressful situations [41].

The current study encountered limitations, including a relatively small sample size and the inability to conduct follow-up on resilience training post-study due to time constraints in project implementation. Hence, future studies are commended to have larger sample sizes with follow-up assessments at 3- and 6-months post-training. Additionally, a separate study could be conducted to train both parents with long-term follow-up evaluations to assess the impact of these interventions on enhancing parental and familial well-being.

## Conclusion

Resilience training reduced the level of parental stress and increased the level of hope and resilience in the mothers of the test group. For this reason, resilience training can be used to equip parents, especially mothers who are most involved with children in their growth and development path. The important point is that increasing resilience can lead to increased hope, and increased hope can, in turn, increase the ability to cope with challenges and hardships in mothers. Accordingly, resilience can be a simple but very effective tool for empowering parents in this path. Therefore, it is recommended to use resilience to reduce parental stress and also increase the level of hope and resilience in mothers living with mentally and physically disabled children.

## Data Availability

The datasets used and/or analysed during the current study available from the corresponding author on reasonable request.
